# Inhibition of recurrence of epithelial ingrowth with an amniotic membrane pressure patch to a laser in situ keratomileusis flap with a central stellate laceration: a case report

**DOI:** 10.1186/s12886-016-0291-4

**Published:** 2016-07-18

**Authors:** Kye Yoon Kwon, Yong Woo Ji, Jeihoon Lee, Eung Kweon Kim

**Affiliations:** Department of Ophthalmology, Severance Hospital, Yonsei University College of Medicine, Yonsei-ro Seodaemun-gu, 120-752 Seoul, South Korea; Corneal Dystrophy Research Institute, Severance Biomedical Science Institute, Institute of Vision Research, Yonsei University College of Medicine, Seoul, South Korea

**Keywords:** Laser in situ keratomileusis, Epithelial ingrowth, Amniotic membrane

## Abstract

**Background:**

Surgical lifting and scraping is a well-known treatment for epithelial ingrowth, but treatment for epithelial ingrowth on the centrally perforated laser in situ keratomileusis (LASIK) flap has not been well studied.

**Case presentation:**

We present a patient who had epithelial ingrowth to the backside of the flap through a central LASIK flap laceration with a stellate shape. The patient had undergone uncomplicated bilateral LASIK surgery 3 years before the trauma. Because the epithelial ingrowth was suspected during the first visit 2 weeks after trauma, and definite epithelial ingrowth was noted during the additional 2 week observation period, the ingrown epithelial tissue was removed mechanically with a number 15 blade after lifting of the flap 4 weeks after the trauma. An amniotic membrane overlay was applied over the cornea and was sutured tightly to the episclera to firmly press down the flap to the remaining posterior stroma, to prevent growth of the epithelium again to the backside of the flap. At the last follow-up visit, 5 months after surgery, the patient’s visual acuity remained stabilized with no sign of recurrent epithelial ingrowth.

**Conclusion:**

These results showed that an amniotic membrane patch can be a useful adjuvant in the treatment of epithelial ingrowth, even on the central stellate laceration of the LASIK flap over the visual axis.

## Background

Epithelial ingrowth after laser in situ keratomileusis (LASIK) has been reported to occur in 0–20 % of cases [1–5]. Surgical lifting and epithelial ingrowth scraping is a well-known treatment for clinically significant epithelial ingrowth with or without adjunctive treatments such as ethanol, mitomycin C, phototherapeutic keratectomy, or suturing of the flap [6–9]. Treatment for epithelial ingrowth on the centrally perforated LASIK flap, however, has not been comprehensively reported.

Human amniotic membrane has been used for ocular surface reconstructions to treat various ocular surface disorders such as chemical burns, symblepharon, persistent epithelial defects, and sterile corneal ulcerations [10, 11]. We have successfully applied an amniotic membrane to inhibit the recurrence of the epithelial ingrowth in linearly lacerated LASIK flap cases [12].

When the stellate laceration occurs at the central LASIK flap, suturing the cornea, including the central flap, would leave a scar from the suture track. Epithelial ingrowth through the central stellate wound can recur even with early scraping of epithelium from the posterior surface of the flap and the anterior surface of the posterior remaining stromal bed. To inhibit the regrowth of the epithelium toward the space where the ingrown epithelia has occurred, tight apposition of the posterior surface of the flap to the posterior remaining stromal bed is necessary.

In the following report, we present a case of central laceration of the flap with a stellate shape, through which epithelial ingrowth occurred during the 4 weeks after trauma. Flap lifting, mechanical epithelial scrapping out, and a pressure patch using an amniotic membrane graft were used to achieve a successful outcome.

## Case presentation

A 33-year-old male was referred to our clinic for corneal opacity with decreased visual acuity in the left eye after injury by a sharp pebble 2 weeks earlier. He had undergone uncomplicated bilateral LASIK surgery 3 years before the trauma, and good visual acuity had been maintained. At the time of the injury, he was abroad and treated with bandage contact lens (BCL) and anti-inflammatory eyedrops by the local physician.

**Fig. 1:**
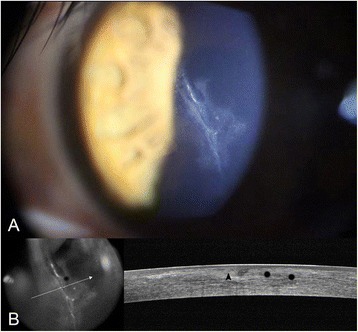
**a** Slit-lamp photograph of the left eye taken at the presentation at our clinic, showing a 5.0 mm long vertical lacerated LASIK flap involving the centre of the pupil. **b** Anterior segment optical coherence tomography (OCT) showing separation of Bowman’s layer after laceration (*black arrowhead*). Epithelial ingrowth from the flap margin to the backside of the flap was suspected, but was not definitely confirmed using OCT (*black asterisk*)

On examination at our clinic, the uncorrected distance visual acuity (UCVA) was 20/60 and the best corrected visual acuity (BCVA) with a spherical correction of −1.50 diopter was 20/40 in the left eye, while UCVA of the right eye was 20/20. Slit-lamp examination of the left eye revealed a 5.0 mm long vertical and central stellate-shaped laceration crossing the centre of the pupil with haze around the lacerated wound. There were no signs of infection. Anterior segment Fourier-domain optical coherence tomography (FD-OCT) (RTVue-100; Optovue, Fremont, CA, USA) showed a laceration with perforation of the LASIK flap and a superficial laceration on the posterior remaining stroma. Epithelial ingrowth to the backside of the flap through the flap margin was suspected, but was not definite in shape (Fig. 1). Gatifloxacin (0.3 %) (Gatiflo^®^; Handok, Chungbuk, Korea) and 1 % prednisolone acetate (Pred Forte^®^; Allergan, Irvine, CA, USA) drops were prescribed for the left eye, four times daily.

**Fig. 2:**
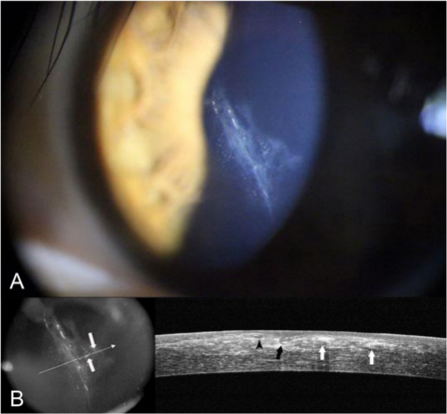
After 2 weeks, epithelial ingrowth and epithelial cysts (*white arrows*) were noted in the slit-lamp photograph **a** and using OCT **b**. The lacerated margin of Bowman’s layer of the LASIK flap (*black arrowhead*) and lacerated and bent margin of the opposite LASIK flap (*black arrow*) were noted using OCT

**Fig. 3:**
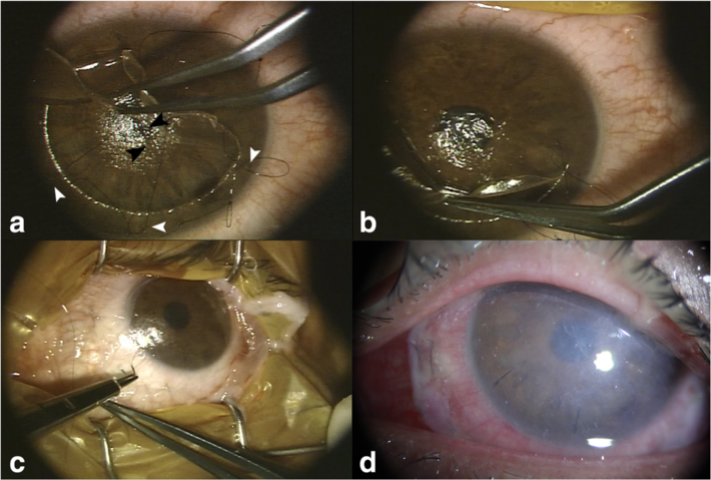
Intraoperative and postoperative photographs showing amniotic membrane pressure patch for inhibition of epithelial ingrowth after LASIK flap laceration. **a** After introducing preplaced sutures along the flap margin at 4, 6, and 8 o’clock (*white arrowheads*), half of the LASIK flap including lacerated central portion, was lifted gently with forceps. Shallow three-branched lacerations were noted on the posterior remaining stromal surface, corresponding to the LASIK flap laceration area (*black arrowheads*). The epithelium was scraped out from both the posterior surface of the flap and the surface of posterior remaining stromal bed with a #15 Bard-Parker blade. **b** The flap was placed back to its original position by tightening the preplaced sutures. **c** Amniotic membrane was tightly sutured with 10–0 nylon to the episclera to cover the entire cornea with the amnion side facing the down position, and the same suturing procedure was repeated with the application of another larger amniotic membrane. **d** The amniotic membrane patch maintained the tightness with pressing down the flap firmly for 5 days after its application, and was removed 10 days after application

Two weeks after the first visit to our clinic, epithelial ingrowth was observed with more notable epithelial cysts (Fig. 2a) with a decreased UCVA of 20/200. After placing preplaced sutures along the flap margin at 4, 6, and 8 o’clock positions to secure the pre-lifting edge, half of the LASIK flap including the lacerated central portion, was lifted gently with forceps (Fig. 3a). The opaque material with several white cystic dots around the laceration (Fig. 2a) was determined by slit-lamp examination to be a grown epithelial sheet. Shallow three-branched lacerations were also noted on the posterior remaining stromal surface, corresponding to the LASIK flap laceration area (Fig. 3a). The epithelium was scraped out from both the posterior surface of the flap and the surface of posterior remaining stromal bed with a #15 Bard-Parker blade. After irrigation of the corneal flap with balanced salt solution (BSS), the flap was placed back to its original position by tightening the preplaced sutures (Fig. 3b). Additional interrupted sutures were placed along the flap margin. Suturing the central cornea near the pupil was avoided, because the laceration was over the pupil. Instead, preserved amniotic membrane was placed over the entire cornea with the amnion side facing the down position and was tightly secured with 10–0 nylon sutures to the episclera, with a tension facilitating pressing down of the corneal flap to the posterior remaining stroma (Fig. 3c). The same suturing procedure was repeated with the application of another larger amniotic membrane to reinforce the pressure force of the LASIK flap to the posterior remaining stroma, and to lengthen the amniotic membrane survival time. We used amniotic membrane frozen from fresh foetal membrane (both amnion and chorion) from the Severance Tissue Bank, because the lyophilized one would be torn off especially at the suture site when the membrane stretched for suturing. Ofloxacin (0.3 %) (Ocuflox; Samil Pharmaceutical, Seoul, Korea) eye drops were prescribed four times a day. The amniotic membrane patch maintained its tightness and firm pressure over the flap firmly 5 days after its application (Fig. 3d), and was removed after 10 days postoperatively.

**Fig. 4:**
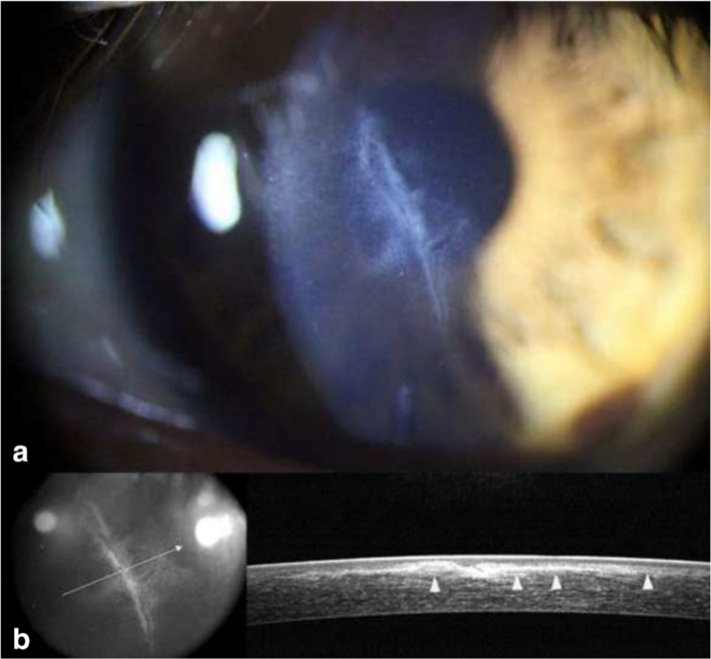
One month after surgery, mild stromal fibrosis (white arrowheads) was noted along the interface between the LASIK flap and posterior remaining stroma in **a** a slit-lamp photograph, and **b** using OCT

**Fig. 5:**
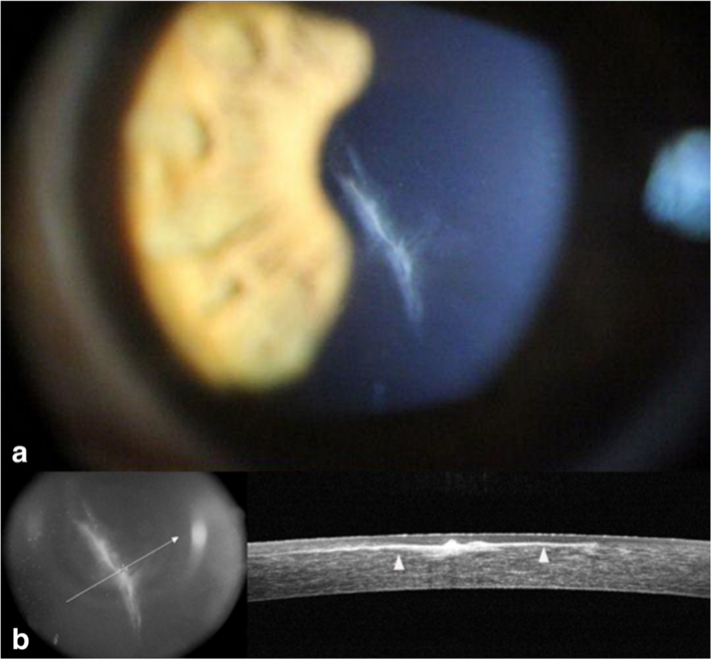
Five months after surgery, there was no sign of recurrent epithelial ingrowth. Fibrotic tissue (*white arrowheads*) was noted along the interface between the LASIK flap and the posterior remaining stroma in **a** the slit-lamp photograph and in **b** using OCT

After removal of the amniotic membrane patch, corneal opacity from the epithelial ingrowth disappeared, with only a small amount of linear opacity remaining along the lacerated LASIK flap margin. Corneal sutures made at the LASIK flap margin were removed 1 week after the amniotic membrane patch removal. At the follow-up visits 1 and 5 months after the epithelial removal, the patient’s UCVA and BCVA with a spherical correction of +0.50 diopter were maintained at 20/40 and 20/20 in the left eye, respectively. There was no sign of recurrence of epithelial ingrowth (Figs. 4 and 5).

## Discussion

In treating a stellate-shaped lacerated LASIK flap wound over the pupil accompanying epithelial ingrowth, there is no new technique for attaching the flap to the posteriorly remaining stroma that results in complete inhibition of epithelial ingrowth. We have previously reported the effect of an amniotic membrane pressure patch for the management of the epithelial ingrowth accompanied by the LASIK flap trauma. The patients with epithelial ingrowth after an intraoperative corneal defect could be treated successfully by using an amniotic membrane as a biological pressure patch [13]. In patient who had a broadly flipped LASIK flap after trauma accompanied by epithelial ingrowth, the amniotic membrane was used as an overlay over the cornea as a biological pressure patch after removing the ingrown epithelial sheet and repositioning of the flap [12]. In the present study, the stellate-shaped laceration wound of the LASIK flap was located on the visual axis. Epithelial ingrowth through the lacerated wound of the flap to the interface between the LASIK flap and the posteriorly remaining stroma progressively interfered with vision. The operation was performed 1 month after the trauma, because the patient presented to us 2 weeks after injury and another 2 weeks were required to confirm the progression of the epithelial ingrowth. When epithelial removal is performed later, the surgical approach must be adjusted to allow more time for stromal remodeling by the epithelium [14]. Our present case using an amniotic membrane patch maintained a stable vision of 20/20 without any signs of recurrence of epithelial ingrowth for 5 months.

Similar to our case, Meskin et al. reported the case of central epithelial ingrowth caused by perforating corneal injury [15]. Unlike our treatment, they sutured the central LASIK flap in a similar manner commonly carried out when the lesion is located peripherally and placed BCL at the end of procedure. They reported disappearance of epithelial ingrowth with no recurrence over 1-year follow-up, however, final astigmatism of 1.25 diopter remained. Previous reports demonstrated that flap margin suturing for epithelial ingrowth is a safe technique with no evidence of flap striae or significant change in spherical equivalence in manifest refraction [6, 14]. However, direct suture method on the central flap lesion can cause deterioration of visual axis, postoperative astigmatism, and iatrogenic creation of epithelial cell tract [15, 16].

Upon the general management of epithelial ingrowth, modalities used to facilitate flap adherence and prevent fistulous epithelial tracts include sutures, fibrin glues on peripheral edge, and BCL treatment. Inhibition of offending epithelial ingrowth at the flap edge would be very important when LASIK flap is intact and dislocated [17]. However, in present report, the lacerating corneal injury with stellate shape caused epithelial ingrowth directly from the central entry point without peripheral flap dislocation. Furthermore, the lacerated lesion also involved the posterior remaining stromal bed. Therefore, it is essential to press down the central portion of the cornea for proper management of the our case. It is obvious that BCL application has beneficial effects on wound healing, however, BCL is difficult to press the center of flattened cornea by LASIK effectively since it is made for the naïve cornea. In fact, the patient had been treated with BCL already before the patient visited to our clinic and epithelial ingrowth occurred with deteriorating vision within 2 weeks after BCL use.

Because of the difficulty in preventing recurrence of epithelial ingrowth, various removal methods of epithelial cells have been proposed, that include simple scraping, excimer laser phototherapeutic keratectomy, and the use of adjuncts such as cryotherapy, alcohol, mitomycin C, or sutures. The recurrence rate after scraping alone has been reported as 44 % [5]. Adjunctive treatments to lower this recurrence rate, however, did not result in a significant difference compared to the results of simple scraping. The use of 70 % isopropyl alcohol showed no additional benefit over simple mechanical scraping in the removal and prevention of epithelial ingrowth recurrence following LASIK [7]. Moreover, compared with mechanical debridement, some reports reported that alcohol can induce keratocyte death and increase inflammation [18]. Mitomycin C can inhibit corneal wound healing [19], and phototherapeutic keratectomy can induce irregular astigmatism and unpredictable shifts in refraction [20]. These side effects prohibited their use in treatment of the stellate-shaped laceration of the LASIK flap.

The observed clinical effects of an amniotic membrane include facilitation of epithelialization, maintenance of a normal phenotype, and reduction of inflammation, vascularization, and scarring [21]. These biological characteristics may explain why the amniotic membrane contributed to blocking the recurrence of epithelium ingrowth in our case, although the patient had a complicated laceration with torn flaps in three directions. The elastic amniotic membrane, after being tightly sutured to the episclera with expansion, pressed down the lacerated flap margin firmly to the posterior remaining stromal surface to inhibit epithelial ingrowth to the LASIK flap interface, even though a combined stromal laceration induced irregularity at the LASIK flap interface.

## Conclusion

Application of an amniotic membrane patch can be a useful adjuvant in the treatment of a stellate-shaped central LASIK flap laceration when epithelial ingrowth occurs over the visual axis.

## Abbreviations

BCL, bandage contact lens; BCVA, best corrected visual acuity; BSS, balanced salt solution; LASIK, laser in situ keratomileusis; UCVA, uncorrected visual acuity
